# Subject: Letter to the Editor regarding “Translation, Cross-cultural Adaptation, and Validation of the Kannada Version of the Exercise Adherence Rating Scale (EARS-Kn) Among Head and Neck Cancer (HNC) Survivors in a Tertiary Care Setup in India” in Integrative Cancer Therapies

**DOI:** 10.1177/15347354251331102

**Published:** 2025-03-29

**Authors:** Chindhu Shunmuga Sundaram

Dear Editor,

I am writing to commend the authors of the study titled “Translation, Cross-Cultural Adaptation, and Validation of the Kannada Version of the Exercise Adherence Rating Scale (EARS-Kn) Among Head and Neck Cancer (HNC) Survivors in a Tertiary Care Setup in India.”^1^ This study represents a significant step in making patient-reported outcome measures (PROMs) more accessible to Kannada-speaking populations and ensuring better adherence assessment among HNC survivors.

Culturally adept PROMs are essential in multinational clinical trials and research, where data from diverse populations must be equivalent and comparable.^2^ With the growing need for PROMs in multiple languages, simple literal translation is insufficient due to linguistic and cultural differences.^3^ Instead, cultural adaptation ensures conceptual equivalence, improving response accuracy and capturing true health-related quality of life differences. Properly adapted PROMs enhance patient engagement, support equitable healthcare decisions, and meet international scientific standards.

However, I would like to highlight the critical importance of incorporating cognitive debriefing interviews with a sample of the target population in the process of cultural and linguistic validation of PROMs, a component that appears to be lacking in this study.^4,5^ Authors have used only a visual analogue scale to assess if the translated version was understandable, if the language used captured the original concepts, and whether items were relevant to their patient population. Based on Beaton et al,^4^ guidelines for the process of cross-cultural adaptation of self-report measures, testing the prefinal version includes study participants completing the PROM and being interviewed to probe about what they thought was meant by each item and the chosen response. Cognitive debriefing interviews serve as a valuable method to ensure that translated items are not only linguistically accurate but also conceptually equivalent to the original version. Without this step, there is a risk that the translated version may not fully capture the intended meanings, leading to potential misinterpretations by the target population.^2^

By integrating cognitive debriefing interviews, researchers can identify, and address issues related to ambiguous wording, cultural nuances, and the relevance of specific items in the local context. This step is particularly crucial in populations with varying levels of literacy and health literacy, as is often the case among HNC survivors in India. The inclusion of cognitive debriefing interviews would enhance the overall validity and reliability of the EARS-Kn, ensuring that it accurately measures exercise adherence within this demographic.

Additionally, the study has certain limitations that should be acknowledged. The sampling method did not focus on reflecting the range and diversity of the sample, including characteristics such as age, gender, educational qualification, HNC type, disease stage, treatment type and status, and substance use (tobacco and alcohol). Since the EARS-Kn is being validated for a diverse population, ensuring representation across key demographic variables ensures that the PROM accurately captures adherence behaviours across all relevant subgroups, preventing measurement bias.

Furthermore, the sample size was not determined following the rule of thumb sample size calculation for quantitative studies, which recommends 10 participants per item in a questionnaire (which indicates a minimum of 160 participants in this study). Another point of concern is the inclusion criterion specifying a histologically proven diagnosis of HNC Stages III and IV (IVa and IVb). Exercise has been shown to benefit survivors across all stages of HNC, including those in earlier stages. Restricting the study population to advanced-stage patients limits the applicability of the findings and overlooks the potential benefits that exercise interventions may offer to individuals at different stages of the disease trajectory. Addressing these limitations in future research would further enhance the generalizability and robustness of the findings.

I appreciate the authors’ efforts in translating and validating this important PROM and encourage future research to adopt a more comprehensive approach by including cognitive debriefing interviews in the cross-cultural adaptation process. Such an approach would further strengthen the applicability of PROMs across settings.

Thank you for the opportunity to provide feedback on this valuable study. I look forward to future advancements in this field.


Chindhu Shunmuga Sundaram, PhD The University of Sydney, Faculty of Science, School of Psychology, Centre for Medical Psychology and Evidence-based Decision-making, NSW, Australia

